# Meropenem therapy in extracorporeal membrane oxygenation patients: an ongoing pharmacokinetic challenge

**DOI:** 10.1186/s13054-015-0953-2

**Published:** 2015-06-22

**Authors:** Patrick M. Honore, Rita Jacobs, Inne Hendrickx, Elisabeth De Waele, Viola Van Gorp, Herbert D. Spapen

**Affiliations:** ICU Department, Universitair Ziekenhuis Brussel, Vrije Universiteit Brussel, Brussels, Belgium

We agree with the recent commentary of Gonçalves-Pereira and Oliveira refuting a ‘one size fits all’ paradigm of antibiotic dosing in patients subjected to extracorporeal circulation [1] but want to focus somewhat further on meropenem pharmacokinetics (PK).

Meropenem has optimal bactericidal activity if plasma levels remain above the minimum inhibitory concentration of the pathogen for at least 40 % of the dosing interval. Still, many patients with severe sepsis do not attain this PK target, have unpredictable PK changes or show large individual and inter-patient variability in distribution volume and clearance when treated with recommended meropenem doses [2, 3].

Meropenem is degraded and significantly sequestered in the extracorporeal membrane oxygenation (ECMO) circuit after 4 to 6 h of treatment. ECMO also induces a systemic inflammatory response which, independently of underlying sepsis, impairs normal meropenem PK [4]. Thus, optimization of meropenem treatment during ECMO requires either more frequent dosing, a dose increase, or prolonged infusion. Ideally, meropenem should be infused continuously over 24 h but, due to its relative instability at room temperature, only a 3-hour infusion is safely feasible. We suggest to administer a 3-hour infusion of 2 g meropenem every 6 h [4]. Future adaptations must be anticipated. We refer to the recently proposed concept of ‘dose modulation’, which concentrates the largest weight of antibiotics front-end when the microbial load is highest and gradually reduces antibiotic dose as sepsis improves [5]. This would imply increasing the loading dose of meropenem (4 g?) and ensuring an adequate maintenance dose guided by PK parameters.

## Abbreviations

ECMO: Extracorporeal membrane oxygenation
PK: Pharmacokinetics
